# Antimicrobial and antibiofilm activity of a novel bacteriophage endolysin (LysSW21) against methicillin-resistant *Staphylococcus aureus*

**DOI:** 10.1186/s12866-026-04916-w

**Published:** 2026-04-16

**Authors:** Sadegh Ranjbari, Narjes Noori Goodarzi, Maryam Banar, Farzad Badmasti, Mir Saeed Yekaninejad, Mohammad Reza Pourmand

**Affiliations:** 1 Department of Pathobiology, School of Public Health, and Recombinant Vaccine Research Center, Tehran University of Medical Sciences, Tehran, Iran; 2 Department of Pathobiology, School of Public Health, and Biotechnology Research Center, Tehran University of Medical Sciences, Tehran, Iran; 3 Department of Bacteriology, Pasteur Institute of Iran, Tehran, Iran; 4 Department of Epidemiology and Biostatistics, School of Public Health, Tehran University of Medical Sciences, Tehran, Iran

**Keywords:** Bacteriophage therapy, Methicillin-resistant *Staphylococcus aureus*, Biofilm, Endolysin, LysSW21, Antimicrobial resistance

## Abstract

**Background:**

The rise of antibiotic-resistant bacteria, particularly methicillin-resistant *Staphylococcus aureus* (MRSA), poses a critical global health challenge. Endolysins, which enzymatically degrade bacterial cell walls, represent a promising class of antimicrobial agents. This study aimed to express and characterize the endolysin LysSW21, derived from the previously characterized Staphylococcus phage vB_SauR_SW21, and evaluate its antibacterial and antibiofilm activities against MRSA strains.

**Results:**

*In silico* analysis of LysSW21 revealed a modular structure comprising an N-terminal CHAP catalytic domain (residues 20–107) and a C-terminal SH3b cell wall-binding domain (residues 164–228). The recombinant protein was successfully expressed in *Escherichia coli* BL21 (DE3) and purified. LysSW21 demonstrated potent bactericidal activity against reference MRSA strains (MIC/MBC: 25 μg/mL) and a clinical isolate from diabetic foot ulcer (MIC/MBC: 12.5 μg/mL). Time-kill assays revealed rapid, concentration-dependent killing, with complete eradication of planktonic MRSA within 75 min at 2 × MIC (50 μg/mL) and within 105 min at MIC (25 μg/mL). The enzyme exhibited remarkable stability across a broad temperature range (4–70°C) and pH range (4.5–10.5), maintaining 4–5 log₁₀ reductions in bacterial viability. Importantly, crystal violet assay and field emission scanning electron microscopy confirmed concentration-dependent antibiofilm activity of LysSW21, with 70–80% biofilm disruption at 4 × MIC against MRSA strains.

**Conclusions:**

LysSW21 exhibits potent bactericidal activity against planktonic MRSA and effectively disrupts biofilms with high stability under diverse environmental conditions. These properties position LysSW21 as a promising candidate for further preclinical development against biofilm-associated MRSA infections. Future studies should evaluate its efficacy and safety *in vivo*.

**Supplementary Information:**

The online version contains supplementary material available at 10.1186/s12866-026-04916-w.

## Introduction

Methicillin-resistant *Staphylococcus aureus* (MRSA) has emerged as a formidable pathogen in healthcare and community settings. This superbug is implicated in a broad spectrum of serious infections, from superficial skin and soft tissue infections to life-threatening conditions such as endocarditis, osteomyelitis, and bloodstream infections [1, 2]. Treatment of MRSA infections has grown increasingly challenging due to the pathogen's rapid development of antibiotic resistance. This crisis has escalated with the emergence of multidrug-resistant (MDR) strains that evade conventional therapies [3]. Recognizing its grave public health threat, the World Health Organization (WHO) classified MRSA as one of the most dangerous antibiotic-resistant bacteria, listing it as a high-priority threat [4]. The clinical and economic burdens are substantial; hospital-treated MRSA infections incur average costs of approximately $22,300 per case and are associated with mortality rates of 12–16%. The global prevalence of MRSA has risen steadily in recent years, with most strains exhibiting broad resistance to β-lactam antibiotics, severely limiting therapeutic options [5]. Compounding this challenge, *S. aureus* biofilm formation represents a critical virulence determinant that substantially increases antibiotic resistance and contributes to persistent infections, including implant-related infections, chronic wound complications, osteomyelitis, cystic fibrosis-associated pneumonia, and endocarditis [6–9].

Bacteriophage therapy has emerged as a promising alternative for combating bacterial infections, particularly due to its ability to bypass conventional antibiotic resistance mechanisms [10–13]. Notably, phage-encoded endolysins represent a particularly attractive antimicrobial strategy against antibiotic-resistant pathogenic bacteria [14, 15].

Endolysins exert their antibacterial effect through enzymatic degradation of peptidoglycan, an essential structural component of the bacterial cell wall. As recombinant proteins, these enzymes offer several therapeutic advantages over conventional antibiotics and whole phage therapy, including rapid and potent bactericidal activity, high target specificity, broad-spectrum lytic activity against pathogenic bacteria, and a favorable safety profile, with no significant toxicity toward human cells reported for related endolysins in preclinical studies [16–18]. However, some endolysins can face challenges such as instability in physiological environments or susceptibility to proteolysis. Furthermore, the C-terminal cell wall binding domain of phage-encoded endolysin mediates rapid kinetics, which is highly specific to the peptidoglycan of bacterial cells and has a low risk of developing resistance [19, 20]. Notably, endolysins have demonstrated particular efficacy against bacterial biofilms, with numerous studies reporting significant disruption of biofilms across various clinical isolates [21].

Building upon our previous characterization of the environmental Staphylococcus phage vB_SauR_SW21 (NCBI accession no. OR683639) [22], and in light of the growing threat posed by MRSA infections, this study aimed to harness the therapeutic potential of phage-derived endolysins. We focused on the recombinant expression and functional characterization of LysSW21, the endolysin encoded by vB_SauR_SW21, with specific emphasis on evaluating its antibacterial efficacy against MRSA, antibiofilm activity, and potential as a novel therapeutic agent.

## Materials and methods

### Bacterial strains, bacteriophages, and growth conditions

All bacterial strains, phage, plasmids, and primers used in this study are detailed in (Supplementary data 1). A clinical MRSA isolate was sourced from our laboratory's microbial collection bank. The clinical MRSA isolate, used in this study, was isolated and ethically characterized in a previous study by our research group [23]. This isolate (from a diabetic foot ulcer) belongs to clonal complex CC8 and exhibits a multidrug-resistant profile, being resistant to oxacillin, cefoxitin, erythromycin, clindamycin, and ciprofloxacin. The original study involving the patient from whom this strain was isolated received ethical approval, and appropriate informed consent was obtained at that time for its use in future research.

Bacterial cultures were grown in lysogeny broth (LB) or on LB agar at 37°C. For long-term storage, strains were preserved in LB broth containing 20% glycerol (v/v) at −80°C.

This study was approved by the Human Research Ethics Committee (HREC) of the School of Public Health, Tehran University of Medical Sciences (Approval Code: 1403.083).

### *In silico* data analysis

The complete genome sequence of Staphylococcus phage vB_SauR_SW21 (GenBank accession number: OR683639.1) was analyzed to characterize its endolysin (LysSW21). The LysSW21 gene sequence was queried against NCBI’s non-redundant protein database using BLASTp (https://blast.ncbi.nlm.nih.gov/Blast.cgi), and homologous phage endolysins from other *Staphylococcus* strains were retrieved. Multiple sequence alignment was performed using the MAFFT web server (https://mafft.cbrc.jp/alignment/server/). A phylogenetic tree was generated from the aligned sequences and visualized using the iTOL database (https://itol.embl.de/). The tertiary structure of LysSW21 was predicted by homology modeling in Swiss-Model using Staphylococcus phage endolysin (PDB: 8H1I) as a template, with subsequent visualization of secondary structural elements (α-helices, β-sheets, and loops) in PyMOL. Functional domains were annotated using the Conserved Domain Database (CDD) (https://www.ncbi.nlm.nih.gov/Structure/cdd/wrpsb.cgi), and physicochemical properties (molecular weight, pI, and instability index) were calculated with the Protparam tool (https://web.expasy.org/protparam).

### Cloning of the LysSW21 expression vector

Phage vB_SauR_SW21 genomic DNA was isolated using a Norgen Biotek kit (following manufacturer’s instructions) with purity and concentration verified by NanoDrop spectrophotometry (Thermo Fisher Scientific). The LysSW21 gene was aumplified via PCR using AccuPower Pfu PCR PreMix with custom primers (Supplementary data 1) containing 5′ *NcoI*/*XhoI* restriction sites. Thermal cycling conditions comprised: initial denaturation (95°C, 7 min); 30 cycles of denaturation (94°C, 45 s), annealing (53°C, 50 s), and extension (72°C, 50 s); followed by final extension (72°C, 5 min). The 750 bp PCR amplicon was gel-purified (Qiagen PCR Purification Kit), then co-digested with *NcoI*/*XhoI* (Thermo Scientific Inc., USA) alongside the *p*ET-28a (+) vector. Ligation employed T4 DNA ligase (Thermo Fisher Scientific Inc., USA), and the ligation product was transformed into chemically competent *E. coli* DH5α cells via heat shock (42°C, 45 s) [24]. Positive clones were verified by Sanger sequencing (ABI 3730xl DNA Analyzer, Life Technologies), with sequence alignment performed using BLASTN (https://blast.ncbi.nlm.nih.gov/Blast.cgi) and ORF prediction via NCBI ORF Finder (https://www.ncbi.nlm.nih.gov/orffinder/).

### Expression and purification of recombinant endolysin (LysSW21)

The confirmed recombinant plasmid was transformed into *E. coli* BL21 (DE3) competent cells for protein expression. A 1 L LB broth culture containing 50 µg/mL kanamycin (Sigma, Germany) was inoculated with an overnight culture of the transformed *E. coli* BL21 (DE3) cells and grown at 37 °C with shaking until reaching an OD_600_ of 0.5. Protein expression was induced with 0.5 mM isopropyl-β-D-thiogalactopyranoside (IPTG) (Thermo Scientific Inc., USA) at 37 °C at 37 °C for 4 h. The cells were allowed to grow for four more hours before being collected by centrifugation at 5000 rpm for 20 min at 4°C.

The cell pellets were resuspended in SDS-PAGE loading buffer and separated on a 12% polyacrylamide gel, followed by staining with 0.25% Coomassie Brilliant Blue (Sigma Chemical, St. Louis, MO, USA). For Western blotting, proteins were transferred to a PVDF membrane and incubated with an anti-6xHis tag antibody (Invitrogen Life Technologies, USA) at 1:2000 dilution to detect the C-terminal epitope [25]. The pellet was resuspended in five volumes of binding buffer (8 M urea, 500 mM NaCl, 50 mM Tris-base, 10 mM imidazole, pH (7.2–7.4) and sonicated using ten 20-s bursts at 200–300 W, with 20-s cooling intervals between bursts. The supernatant was then mixed with resin and passed through the column. After the liquid flowed through, the resin was washed with four volumes of washing buffer (2 M urea, 500 mM NaCl, 50 mM Tris-base, 20 mM imidazole, pH 7.2–7.4). The column was then treated with elution buffer (2 M urea, 500 mM NaCl, 50 mM Tris-base, 500 mM imidazole, pH (7.2–7.4), slowly pipetted, and incubated at room temperature for 15 min. The column outlet was opened, and the solution containing the target protein was collected. SDS-PAGE was used to verify the size and purity of the protein. The eluted protein was dialyzed against PBS (4°C, 12 h) to remove urea and imidazole, concentrated using centrifugal filters, and quantified by Bradford assay (Bio-Rad) [26]. The recombinant protein was purified with an intact C-terminal 6xHis tag, which was retained for all subsequent functional assays. Aliquots were stored at −80°C.

### Determination of MIC and MBC

The antimicrobial efficacy of LysSW21 was evaluated by determining its minimum inhibitory concentrations (MICs) and minimum bactericidal concentration (MBC) against MRSA using Clinical and Laboratory Standards Institute (CLSI)-approved microbroth dilution assay (performed in technical and biological triplicates). In brief, overnight cultures of MRSA in Mueller–Hinton broth (MHB) were adjusted to 10⁶ colony-forming units per milliliter (CFU/mL), then exposed to serial two-fold dilutions of LysSW21 (0.5–128 μg/mL) in 96-well microplates. After 24-h incubation at 37 °C, MIC values were recorded as the lowest protein concentration that prevented any visible bacterial growth (visual turbidity assessment). For MBC determination, 10 μL aliquots from clear wells were subcultured on BHI agar. The MBC was defined as the lowest concentration achieving ≥ 99.9% reduction in viability compared to untreated controls [27].

### Time-kill assay

The bactericidal kinetics of LysSW21 against MRSA ATCC43300 were evaluated using a time-kill assay by CLSI guidelines. A 0.5 McFarland suspension was prepared from freshly cultured MRSA ATCC43300 colonies and was then diluted 1:10 in MHB to approximately 10⁷ CFU/mL. LysSW21 was added to the diluted bacterial suspension at concentrations of 12.5, 25, and 50 µg/mL, followed by incubation at 37 °C with shaking at 140 rpm. Untreated bacterial samples served as negative controls. At time intervals of 0, 15, 30, 45, 60, 75, 90, 105, and 120 min, samples were collected and serially diluted. A 10 µL aliquot from each dilution was plated on BHI agar plates to determine colony-forming units (CFU). After counting the colonies and calculating CFU/mL, time-kill curves were generated [7].

### Screening of biofilm formation in MRSA strains

Biofilm production by clinical MRSA isolate and reference strain ATCC33591 was evaluated using a modified microtiter plate assay [28]. Overnight cultures in TSB supplemented with 1.5% glucose (w/v) were adjusted to 0.5 McFarland standard (1.5 × 10⁸ CFU/mL) in fresh TSB. Aliquots (100 µL) were transferred to 96-well tissue culture plate (JET BIOFIL, Canada) and incubated statically for 24 h at 37 °C. Post-incubation, non-adherent cells were removed by triple-washing with PBS. The adherent biofilms were fixed with 96% ethanol for 15 min, stained with 1.5% crystal violet, and gently rinsed with tap water to eliminate excess dye. The bound crystal violet was dissolved in 150 µL of 33% glacial acetic acid (v/v), and biofilm biomass was quantified by measuring the absorbance at 550 nm using an ELISA reader (Anthos Labtec, model 22,550). All strains were tested in triplicate across three biological replicates. Based on Stepanović et al*.* [29], only strong biofilm producers were selected for subsequent assays.

### Assessment of LysSW21-mediated MRSA biofilm disruption

The biofilm eradication potential of LysSW21 was quantified using a standardized microtiter plate assay with modifications [30]. Preformed 24-h biofilms (strong producer strain from screening) were treated with serial dilutions of LysSW21, vancomycin (positive control), and PBS (negative control). After 24 h incubation at 37 °C, biofilms were washed three times with PBS to remove non-adherent cells, fixed with 95% ethanol, stained with 1% crystal violet, and dissolved with 33% acetic acid (v/v). The biofilm removal efficacy was calculated using the following formula: (1—OD (sample)/OD (control)) × 100 [31].

### Thermal and pH stability profiling of LysSW21

The recombinant endolysin LysSW21 (25 µg/mL in PBS, pH 7.4) was incubated for 30 min at six temperatures (4, 25, 37, 50, 60, and 70 °C). Following heat treatment, residual lytic activity was determined against MRSA ATCC43300 (10⁷ CFU/mL in TSB) during 24 h incubation at 37 °C with agitation (140 rpm). Bacterial viability was quantified by serial dilution plating (log_10_ CFU/mL).

For pH stability evaluation, LysSW21 (25 µg/mL) was exposed to 50 mM universal Britton-Robinson buffer at varying pH (4.5, 5.5, 7.4, 8.5, and 10.5) for 30 min at 37 °C. After pH neutralization to 7.4, antimicrobial activity was assessed against MRSA ATCC 43300 (10⁷ CFU/mL) for 24 h at 37 °C with agitation (140 rpm). Bacterial viability was quantified by serial dilution plating at 0 h (initial inoculum) and 24 h post-treatment, and results were expressed as Log₁₀ CFU/mL. Untreated controls were included at each pH condition for comparison. All assays included technical triplicates across two biological replicates [32]. Statistical significance between treatment and control groups was determined using two-way ANOVA with Sidak's multiple comparisons test.

### Field emission scanning electron microscopy (FE-SEM)

#### Antibiofilm effect assessment

Preformed MRSA ATCC33591 biofilms were established on sterile glass slides (1 cm2) in 6-well plates (JET BIOFIL) using 3 mL TSB inoculum (1.5 × 10⁷ CFU/mL; 24 h, 37 °C). Following overnight incubation, slides were rinsed three times with PBS to remove planktonic and loosely adherent cells. LysSW21 was then applied at 2 × and 4 × MIC concentrations (1 mL/well), and plates were incubated for an additional 24 h at 37 °C. After treatment, slides were washed twice with PBS, and biofilms were fixed with 2.5% glutaraldehyde for 2 h at 4 °C. Samples underwent sequential dehydration in ethanol gradients (50–100%, 15 min per step), air-dried, and sputter-coated with gold before FE-SEM imaging (MIRA3, TESCAN, Czech Republic) [33].

#### Antibacterial effect assessment

Log-phase MRSA ATCC43300 cells were centrifuged, washed, and resuspended in Tris–HCl (pH 7.4). The bacterial suspension was then mixed with either LysSW21 (50 µg/mL) or Tris buffer (as a control). After incubation at 37 °C for 2 h, the bacterial cells were centrifuged for 10 min and washed twice with Tris–HCl (pH 7.4). The cells were fixed with 2.5% glutaraldehyde, washed with PBS, dehydrated using ethanol, and prepared for further analysis [34].

### Statistical analysis

Statistical analyses were conducted using SPSS (v. 27.0, IBM). All data are presented as mean ± standard deviation (SD) from at least three independent experiments. For multi-group comparisons, one-way ANOVA with Tukey’s post hoc test was applied. Two-group comparisons utilized independent samples t-tests with Welch’s correction for unequal variances when appropriate. Statistical significance was defined as *p* < 0.05 for all analyses. For thermal and pH stability assays, data were analyzed using two-way ANOVA with Sidak's multiple comparisons test to compare treated samples versus untreated controls across all temperature and pH conditions, and to assess differences in killing efficacy between different treatment conditions. Statistical significance was defined as *p* < 0.05 [35].

## Results

### Characterization of endolysin LysSW21

**Fig. 1:**
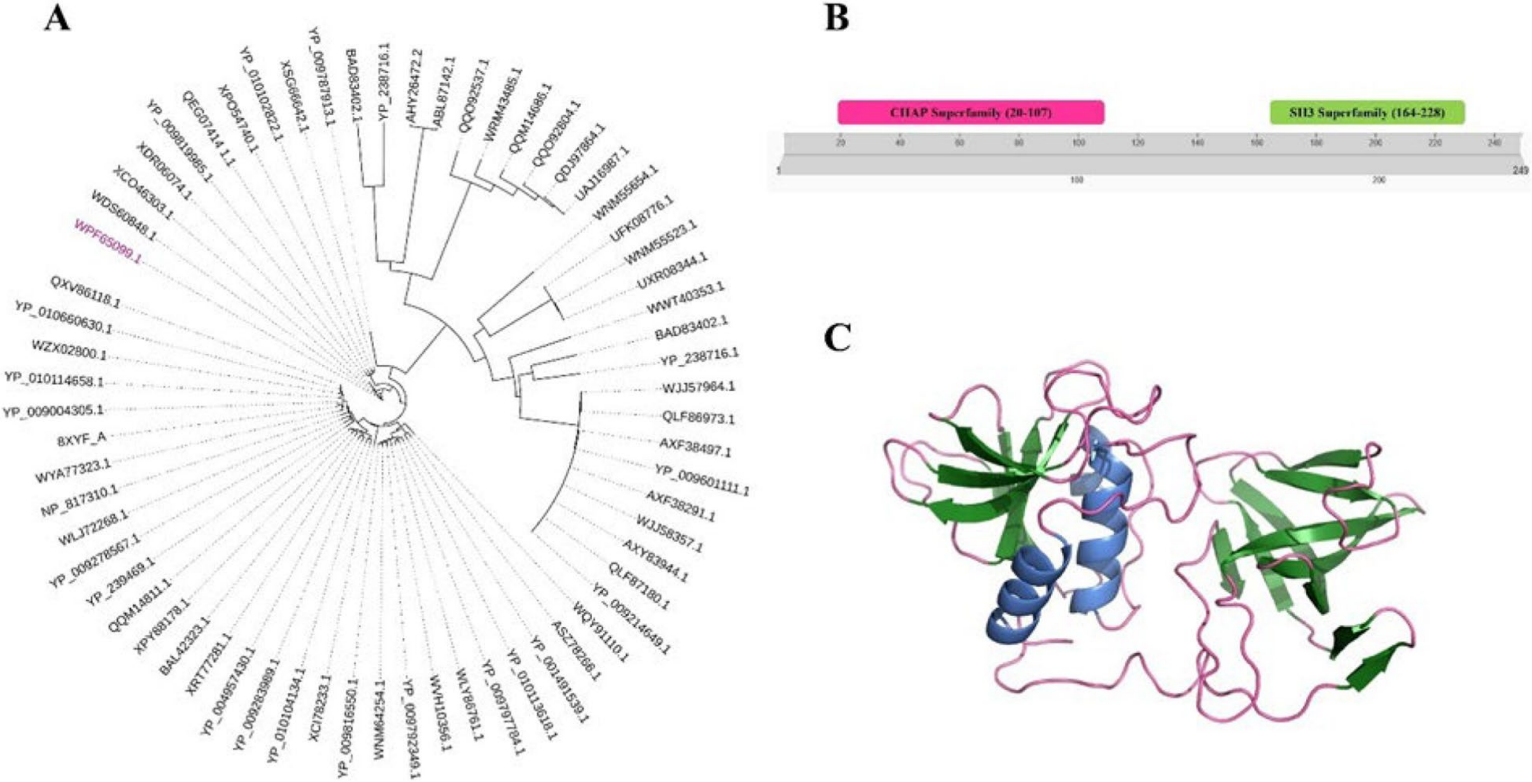
Structural and phylogenetic characterization of LysSW21.** A** The UPGMA phylogenetic tree of LysSW21 and 63 homologous Staphylococcus phage endolysins. **B** Conserved domain analysis showed two conserved domains, including CHAP superfamily and SH3 superfamily, that are located at the N- and C-terminal of the protein, respectively. **C** The 3D structure of the LysSW21. Alpha-helices, beta-sheets, and loops are shown in blue, green, and pink, respectively

Genomic analysis of Staphylococcus phage vB_SauR_SW21 (GenBank: OR683639.1) revealed a potential endolysin designated LysSW21. Comparing the coding sequence of the LysSW21 to the protein database at the NCBI resulted in the identification of 63 known Staphylococcus phage endolysins with partial similarity to our desired protein (Supplementary data 2). The sequence of all proteins were aligned, and a phylogenetic tree was generated (Fig. 1A). The phylogenetic tree showed a high diversity in Staphylococcus phage endolysins, with the highest similarity of LysSW21 to endolysin of Staphylococcus phage Huma from Iran (WDS60848.1) and endolysin of Staphylococcus phage vB_Sau-F2 from Egypt (XCO46303.1). A focused alignment of the catalytic CHAP domain (residues 20–107) with those of key reference staphylococcal endolysins (e.g., LysK, Ply187) revealed a high degree of conservation, including the essential catalytic triad residues characteristic of CHAP-family amidases. Similarly, alignment of the cell wall-binding domain (SH3b, residues 164–228) showed conserved motifs critical for peptidoglycan recognition when compared to homologous domains from other phage lysins targeting *S. aureus*. Conserved Domain analysis revealed the presence of a CHAP superfamily domain on the N-terminal (residues 20–107), which is likely to function as its catalytic region (putative peptidoglycan hydrolase activity) and a SH3 domain on the C-terminal (residues 164–228) of the protein for cell wall binding (Fig. 1B). Moreover, the 3D structure of the protein has been demonstrated in Fig. 1C. In the structural model, the N-terminal CHAP domain and the C-terminal SH3b domain are clearly delineated as distinct structural modules, consistent with the modular architecture typical of phage endolysins. The physicochemical properties of the protein has been presented in (Supplementary data 3).

### Cloning, expression, and purification of endolysin LysSW21

**Fig. 2:**
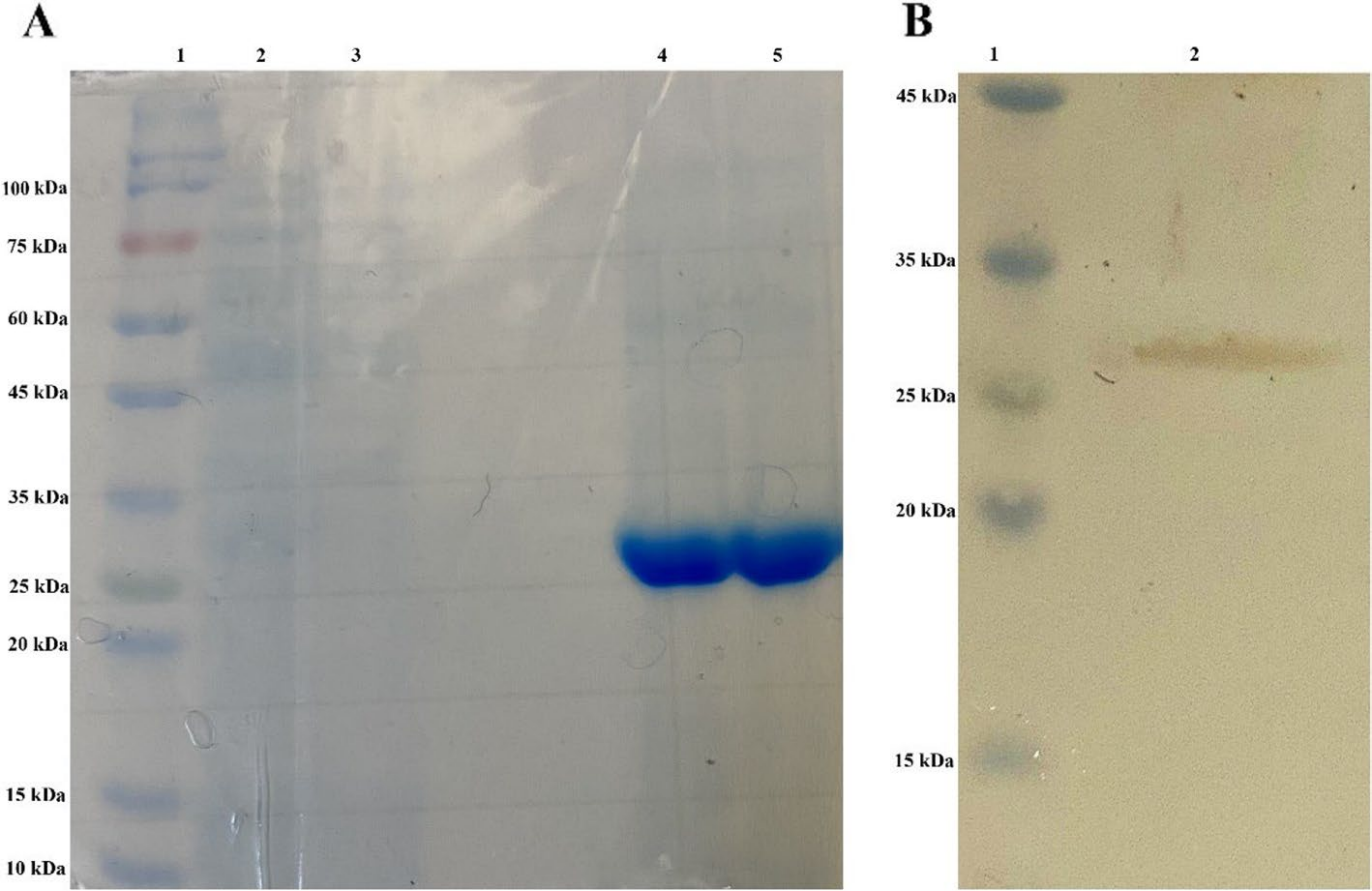
Purification and immunodetection of recombinant LysSW21. **A** SDS-PAGE analysis of LysSW21. Lane 1 displays the molecular size marker; Lane 2 contains the binding sample; Lane 3 shows the wash sample; Lanes 4 and 5 present the purified LysSW21. **B** His-tag Western blot analysis of LysSW21. Lane 1 displays the molecular size marker; Lane 2 shows the purified LysSW21

SDS-PAGE analysis confirmed successful expression and efficient purification of LysSW21 (Fig. 2A). Western blotting using His-tag-specific antibodies detected a protein band at the anticipated molecular weight of ~ 28 kDa (Fig. 2 B). Additionally, the purified LysSW21 had a roughly 490 μg/mL concentration.

### MIC and MBC of LysSW21 against MRSA strains

LysSW21 demonstrated potent bactericidal activity against all tested MRSA strains (MRSA ATCC43300, MRSA ATCC33591, and a clinical isolate), with MIC and MBC values of 25 μg/mL for reference strains (ATCC43300 and ATCC33591) and 12.5 μg/mL for the clinical isolate (diabetic foot ulcer origin). This 2-fold greater potency against the clinical strain suggests either enhanced susceptibility due to altered peptidoglycan cross-linking in the clinical isolate, or strain-specific differences in cell wall accessibility for LysSW21's CHAP domain.

### Time-kill kinetics of LysSW21 against MRSA

**Fig. 3:**
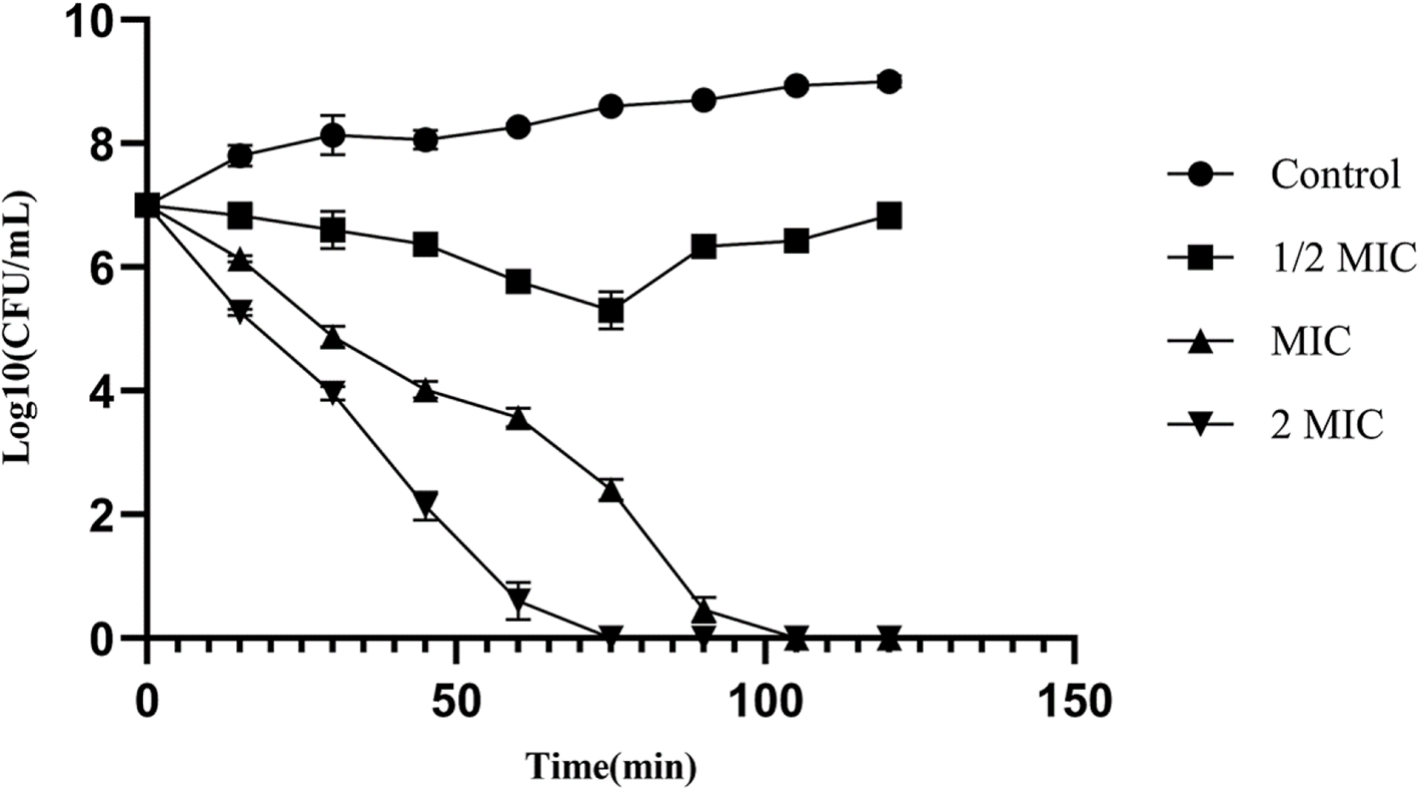
Time-kill kinetics of LysSW21 against MRSA ATCC43300. Bacterial suspensions (~ 10⁷ CFU/mL) were treated with LysSW21 at 1/2 × MIC (12.5 µg/mL), 1 × MIC (25 µg/mL), and 2 × MIC (50 µg/mL). Data represent the mean ± SD from three independent experiments; missing error bars indicate an SD too small to display

The endolysin demonstrated rapid, concentration-dependent killing, achieving complete eradication of the bacterial inoculum within 75 min at 2 × MIC (50 µg/mL) and within 105 min at its MIC (25 µg/mL) (Fig. 3). A sub-inhibitory concentration (1/2 × MIC) produced only a transient reduction in viable counts, with regrowth observed after 75 min. The rapid killing kinetics of LysSW21 provides a key *in vitro* advantage compared to some conventional antibiotics.

### Evaluating the thermal and pH stability of LysSW21

**Fig. 4:**
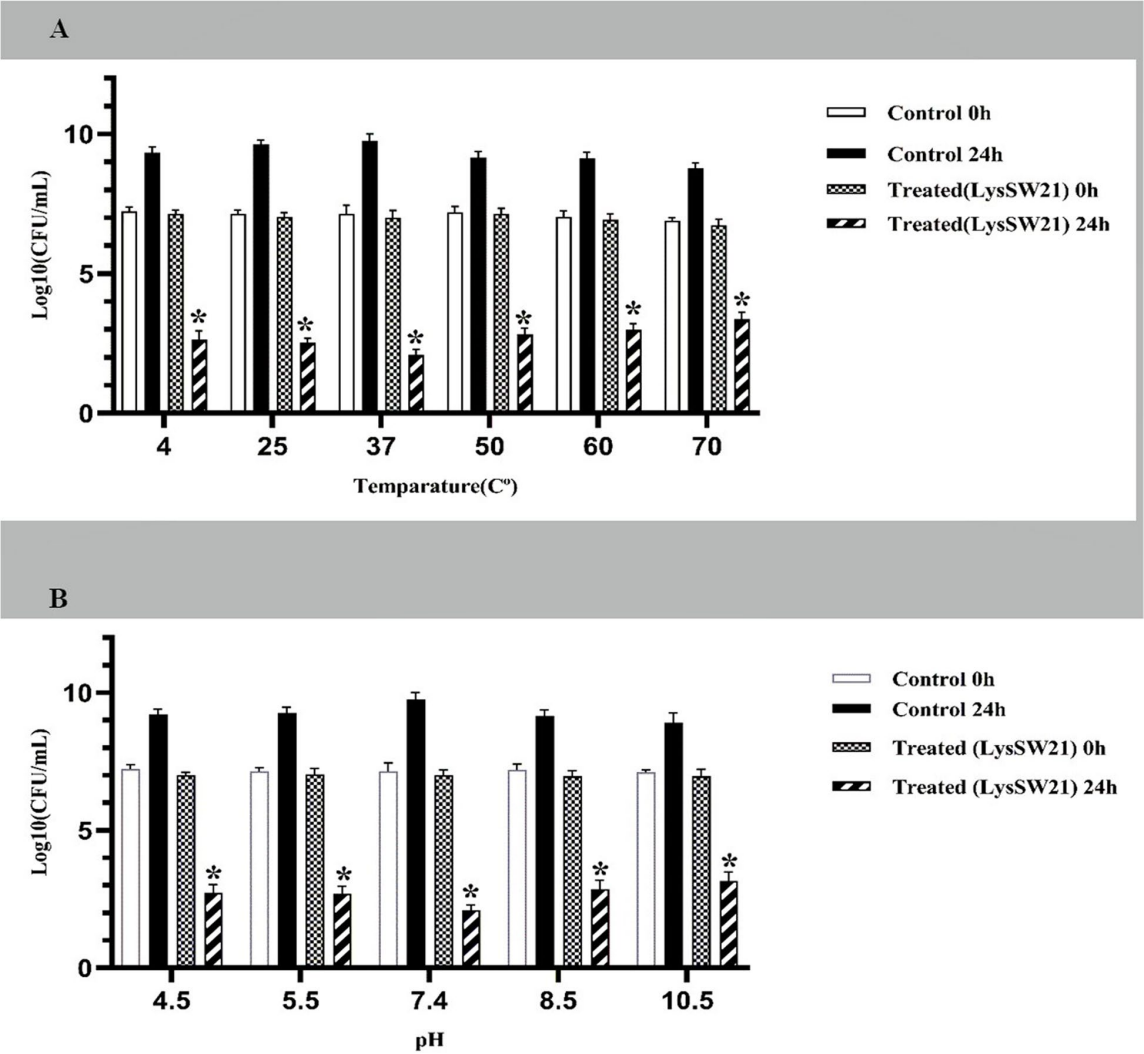
Thermal and pH stability profile of purified endolysin LysSW21. Bacterial suspensions (initial inoculum ~ 10⁷ CFU/mL) were treated with LysSW21 (25 μg/mL) after 30 min pre-incubation under various conditions, and viable counts were determined after 24 h incubation at 37°C. **A** Temperature stability: LysSW21 was pre-incubated at 4–70°C before addition to bacterial cultures. **B** pH stability: LysSW21 was exposed to pH 4.5–10.5, neutralized to pH 7.4, then added to bacterial cultures. Data represent mean ± SD from three independent experiments. Statistical analysis: two-way ANOVA with Sidak's multiple comparisons test; *p < 0.05 for all treated samples versus respective untreated controls

Thermal and pH stability are crucial for ensuring the efficacy of antibacterial agents during storage. LysSW21 exhibited robust stability across a wide temperature range (4–70 °C), maintaining potent bactericidal activity after 30 min pre-incubation at each tested temperature (Fig. 4A). Following 24 h incubation with treated bacterial suspensions, viable counts were reduced by approximately 4–5 log₁₀ compared to the initial inoculum (7 log₁₀ CFU/mL), with no significant difference in killing efficacy across the temperature range (two-way ANOVA, *p* > 0.05). Similarly, LysSW21 retained considerable activity across a broad pH spectrum (4.5–10.5), achieving 4–5 log₁₀ reductions in bacterial viability relative to the initial inoculum (Fig. 4B). Untreated controls at each pH condition showed no significant reduction in viability over 24 h (*p* < 0.05 compared to treated samples). These findings demonstrate that LysSW21 maintains its bactericidal activity under diverse thermal and pH conditions, supporting its potential as a robust antibacterial candidate.

### Antibacterial activity against MRSA biofilms

**Fig. 5:**
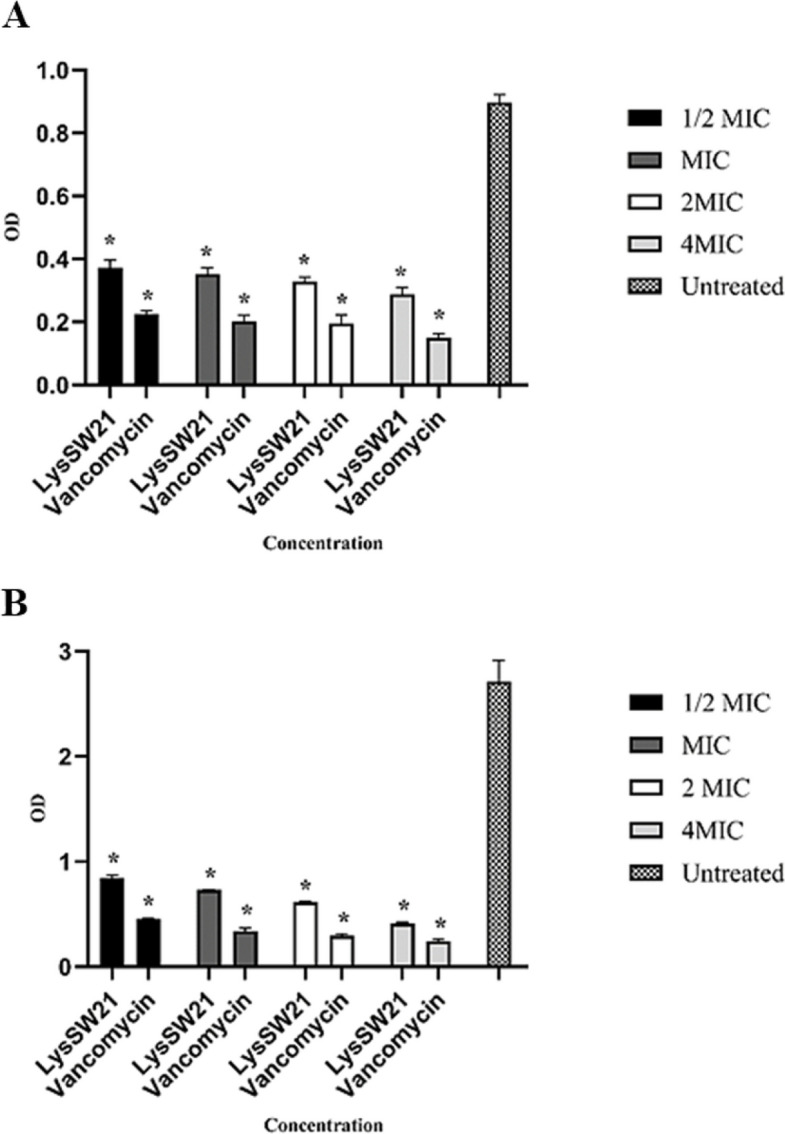
Antibiofilm activity assay. **A** Biofilm-destructive activity of LysSW21 against MRSA ATCC33591. **B** Biofilm-destructive activity of LysSW21 against clinical MRSA. Bars having an asterisk are significantly different from the untreated (ANOVA; *P < 0.001). Data represent the mean ± SD of three independent experiments

The antibiofilm efficacy of LysSW21 was assessed against MRSA strains using the crystal violet assay. For MRSA ATCC33591 (vancomycin MIC = 1 µg/mL), LysSW21 was tested at 12.5–100 μg/mL. For the clinical MRSA isolate (vancomycin MIC = 0.5 µg/mL), LysSW21 was tested at 6.25–50 μg/mL. vancomycin was tested in parallel at concentrations of 0.5 × MIC to 4 × MIC for each respective strain. Compared to untreated controls and vancomycin-treated positive controls, LysSW21 exhibited significant biofilm degradation (*P* < 0.001) in both strains (Fig. 5A, B).

For MRSA ATCC33591 (Fig. 5A), LysSW21 exhibited a significant concentration-dependent effect on biofilm degradation. At 1/2 MIC, biofilm degradation was moderate but still significantly greater than that of the untreated control group. This effect was potentiated at MIC and reached its peak at 4 × MIC, where nearly 70% eradication of the biofilm was achieved. Although the degradative effect of vancomycin was greater, at concentrations equal to or above the MIC, LysSW21 demonstrated relatively good performance.

A similar pattern was observed with the clinical MRSA strain (Fig. 5B). LysSW21 showed significant dose-dependent anti-biofilm activity against the clinical MRSA strain. At 4 × MIC, LysSW21 eradicated approximately 80% of the biofilm.

**Fig. 6:**
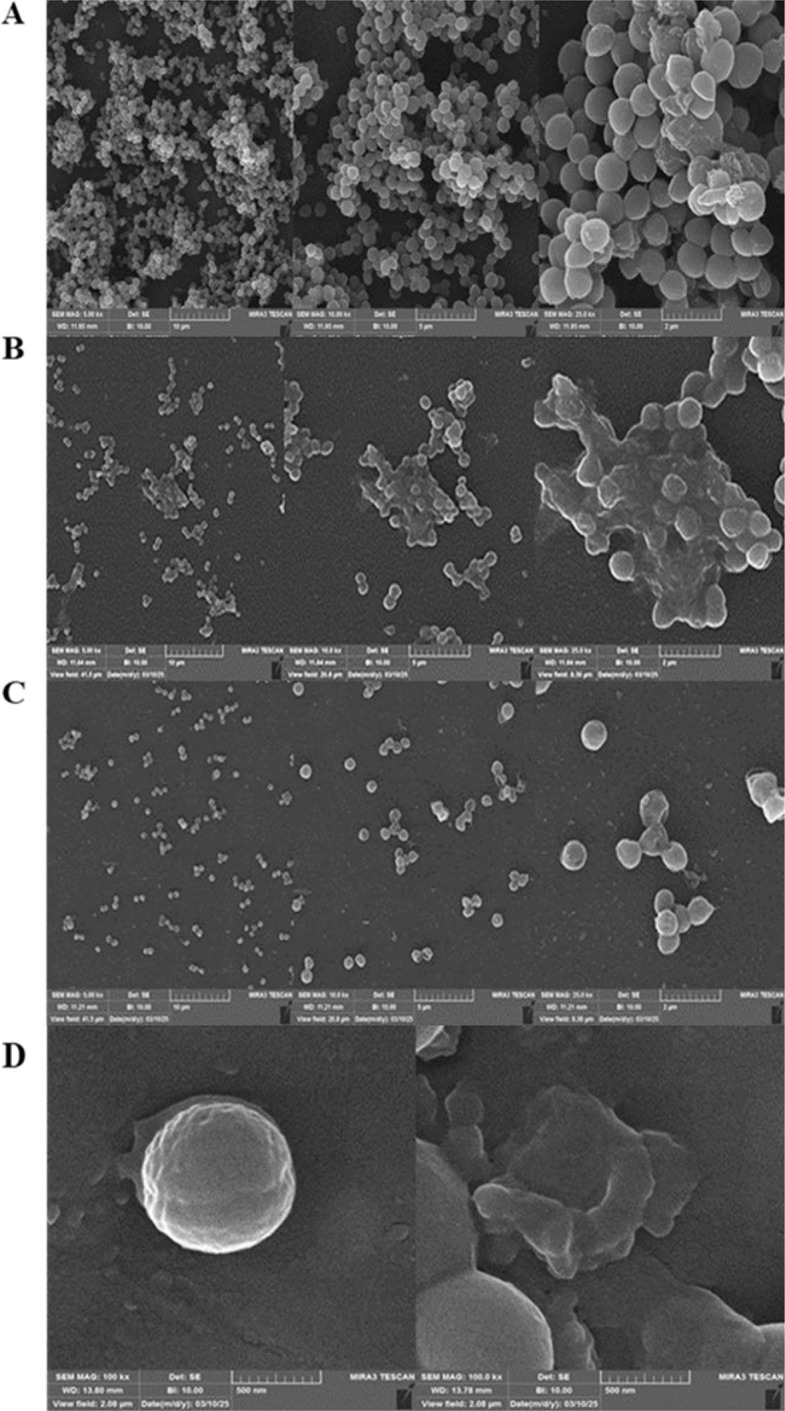
FE-SEM images of biofilm-eradication activity of LysSW21. **A** Untreated biofilm (Control). **B** Biofilm treated with 50 µg/mL LysSW21. **C** Biofilm treated with 100 µg/mL LysSW21. **D** Assessment of the antibacterial activity of LysSW21 (50 µg/mL) against planktonic MRSA cells using SEM micrograph (left panel: untreated MRSA strain; right panel: treated with 50 µg/mL LysSW21 for 2 h). **A**-**C** Magnification of Images were 5.00 k for the left panel, 10.00 k for the middle panel, and 25.00 k for the right panel. **D** Magnification of Images were 100 k for the left and right panels

SEM analysis corroborated these findings (Fig. 6). Untreated cells maintained normal coccal morphology within a dense biofilm matrix, while LysSW21-treated samples (50–100 μg/mL) displayed profound structural damage, including cell wall rupture, surface detachment, and morphological aberrations (Fig. 6A-C). Treatment of planktonic cells with 50 µg/mL LysSW21 also caused clear cell lysis compared to the intact control cells (Fig. 6D).

## Discussion

This study demonstrates that LysSW21, a novel endolysin from Staphylococcus phage vB_SauR_SW21, exhibits potent anti-MRSA activity (MIC/MBC: 12.5–25 μg/mL) with rapid bacteriolysis and significant biofilm disruption. The enzyme maintains exceptional stability across clinically relevant conditions (4–70 °C; pH 4.5–10.5). Structural analysis reveals conserved CHAP (catalytic) and SH3 (binding) domains responsible for its broad-spectrum efficacy. These properties, combined with its ability to penetrate mature biofilms, position LysSW21 as a promising candidate for further development against multidrug-resistant MRSA infections.

The presence of a lysin-like (N-acetylmuramoyl-L-alanine) domain (residues 20–107) in LysSW21 confirms its classification as an amidase, a well-documented family of endolysins that cleave the amide bond between N-acetylmuramic acid and L-alanine in peptidoglycan [36]. Earlier studies have examined the catalytic functions of phage endolysins from other staphylococcal phages, such as LysK [37] and Ply187 [38].

Consistent with previous reports on phage endolysins such as LysK and PlySs2, LysSW21 demonstrated strain-dependent efficacy, showing 2-fold greater potency against a clinical MRSA isolate (MIC/MBC = 12.5 μg/mL) compared to reference strains [39, 40]. This enhanced activity likely reflects optimal compatibility with the clinical strain's peptidoglycan architecture. The observed potency is comparable to other well-characterized endolysins, such as LysGH15 (MIC 12.5–50 μg/mL) [41], and aligns with the bacteriolytic mechanism confirmed by our SEM analysis, which revealed characteristic peptidoglycan degradation similar to that induced by LysK and ClyS [42, 43].

For clinical translation, enzymatic stability is as critical as potency. LysSW21 exhibited impressive thermostability (4–70°C) and broad pH tolerance (4.5–10.5), mirroring the robust profile of engineered endolysins like PlySs2 [44, 45]. This stability suggests potential applicability across diverse anatomical sites, from skin to urinary tract, addressing a common limitation of protein-based therapeutics. The exceptional stability of LysSW21 may be attributed, in part, to its high aliphatic index (60.64) and the presence of two predicted disulfide bonds (Cys28-Cys105 and Cys167-Cys226), features that can enhance thermostability and structural integrity under denaturing conditions.

However, despite promising *in vitro* stability, the translational development of endolysins faces challenges not typically encountered with traditional small-molecule antibiotics. As exogenous proteins, they can be susceptible to proteolytic degradation, rapid renal clearance, and neutralization by the host immune system, which may limit their *in vivo* half-life and efficacy. Furthermore, formulation for systemic delivery and potential immunogenicity with repeated dosing require careful investigation [43].These hurdles underscore the importance of the favorable stability profile observed for LysSW21 *in vitro* (Fig. 4) as a foundational characteristic. Future work must therefore include pharmacokinetic and immunogenicity studies in animal models to determine whether this stability translates into a sufficient therapeutic window *in vivo* [43].

To further contextualize its therapeutic potential within this framework, LysSW21 can be compared to endolysins in advanced clinical development, such as exebacase (CF-301) and tonabacase (SAL200/N-Rephasin®). These candidates have demonstrated significant efficacy in clinical trials against *S. aureus* bacteremia, navigating the challenges described above. LysSW21 shares their key advantageous features: rapid, concentration-dependent killing (complete eradication within 105 min at MIC), potent biofilm disruption, and considerable environmental stability. A distinguishing feature of LysSW21 is its derivation from the novel phage vB_SauR_SW21, which may confer unique cell wall binding specificity. Its compelling *in vitro* profile supports its development as a promising candidate, potentially for use as a monotherapy or as a synergistic adjuvant to conventional antibiotics, as demonstrated by chimeric endolysins like ClyS [42].

Biofilm formation represents a critical virulence determinant in MRSA, facilitating chronic infections and conferring antibiotic resistance [46]. While vancomycin remains a last-line treatment, its effective biofilm-disrupting activity often requires concentrations significantly above the MIC, which can be associated with nephrotoxicity and may promote resistance [47, 48]. These limitations underscore the need for novel, targeted antibiofilm strategies.

Recombinant endolysins have been studied primarily for their planktonic antimicrobial effects, but a growing body of evidence supports their role as antibiofilm agents [18, 49, 50]. Their mechanism involves direct bacteriolysis and disruption of the biofilm’s extracellular polymeric substance (EPS) matrix [18, 49].

Our findings demonstrate that LysSW21 exhibits substantial, concentration-dependent antibiofilm activity. While vancomycin (50 µg/mL) showed strong biofilm disruption under our assay conditions, LysSW21 achieved considerable biofilm reduction (~ 70–80%) at 4 × MIC against both reference and clinical MRSA strains. This activity is significant, as it demonstrates the enzyme’s ability to penetrate the biofilm matrix and directly cleave peptidoglycan, thereby destabilizing the structural framework of bacterial communities [51]. The marginally increased resistance of the clinical isolate at sub-MIC levels may be attributed to strain-specific variations in EPS composition [52], an effect overcome at higher concentrations.

The observed antibiofilm potency of LysSW21 aligns with reports for other promising endolysins. For instance, LysSYL has demonstrated strong activity against *S. aureus* biofilms [35]. The level of biofilm reduction achieved by LysSW21 at elevated concentrations positions it among other potent endolysins reported in the literature. This performance may be attributed to its optimized enzymatic domain architecture, which likely enhances peptidoglycan hydrolysis and biofilm matrix penetration. These results, combined with its favorable stability profile, support the further investigation of LysSW21 as a promising candidate for targeting biofilm-associated MRSA infections.

Time-kill assays revealed that LysSW21 exhibits rapid bactericidal activity, completely eradicating MRSA within 75 min at 2 × MIC. This rapid killing kinetics could be advantageous in rapidly reducing bacterial load *in situ*. Importantly, the rapid bacteriolysis mediated by endolysins and their targeting of conserved peptidoglycan structures are associated with a low risk of resistance development, a significant drawback of conventional antibiotics such as vancomycin [53].

LysSW21 demonstrates considerable promise as a novel antimicrobial agent, combining rapid bactericidal activity, potent biofilm degradation, and remarkable environmental stability. Unlike conventional antibiotics that often fail against biofilm-related infections, this endolysin effectively targets MRSA in challenging *in vitro* scenarios. Crucially, by specifically cleaving highly conserved peptidoglycan bonds, LysSW21 presents a theoretically lower risk of resistance development compared to traditional antimicrobials [39].

While our *in vitro* results demonstrate the therapeutic potential of LysSW21, comprehensive preclinical safety assessments, including cytotoxicity profiling against human cells and pharmacokinetic studies, are essential future steps before clinical translation can be considered. Additionally, synergistic approaches combining LysSW21 with other antimicrobial agents or biofilm-disrupting treatments may enhance its efficacy against complex infections [54]. Furthermore, while promising against the strains tested, the antimicrobial spectrum of LysSW21 should be validated against a larger and more diverse panel of clinical MRSA isolates in future work. Further optimization through protein engineering, including tag removal or the generation of tag-less variants, could further refine its activity and clinical applicability. These development pathways would strengthen its viability as a next-generation anti-MRSA therapeutic.

## Conclusions

The phage-derived endolysin LysASW21 was successfully purified and demonstrated potent lytic activity against MRSA, including clinical isolate, with rapid bactericidal effects observed in time-kill assays. Notably, LysSW21 exhibited robust antibiofilm activity, disrupting preformed biofilms and reducing bacterial viability within the biofilm matrix. The enzyme maintained stability across a wide pH range (4.5–10.5) and temperatures (4–70 °C), underscoring its potential for further development. While these *in vitro* results are promising, future studies must evaluate LysSW21’s efficacy, safety, and pharmacokinetics in animal models to advance its development as a treatment for MRSA infections and biofilm-associated complications. 

## Supplementary Information

Supplementary Material 1.

Supplementary Material 2.

Supplementary Material 3.

## Data Availability

The datasets analysed during the current study are available in the GenBank repository under accession number OR683639.1.
