# Supplementary material for: Antimicrobial and antibiofilm activity of a novel bacteriophage endolysin (LysSW21) against methicillin-resistant Staphylococcus aureus
Source: BMC Microbiol. 2026 Apr 16;26:507. doi: 10.1186/s12866-026-04916-w (PMC13214339; doi:10.1186/s12866-026-04916-w)
Supplement: Supplementary file 1 — Supplementary Material 1. [file 12866_2026_4916_MOESM1_ESM.docx]

**Table 1. Bacterial strains, bacteriophage, plasmids, and oligonucleotide primers used in this study.**

| **Strain, plasmid, phage, or primer** | | **Relevant characteristic(s), description** | **Reference** |
| --- | --- | --- | --- |
|  |  |  |  |
| **Strains** | *S. aureus* | MRSA, ATCC43300 | ATCC |
|  | *S. aureus* | MRSA, ATCC33591 | ATCC |
|  | *S. aureus* | MRSA isolate from diabetic foot ulcer | (23) |
|  | *Escherichia coli* DH5α | Laboratory strain for cloning use | Novagen, WI, USA |
|  | *E. coli* BL21(DE3) | Laboratory strain for protein expression | Novagen, WI, USA |
| **Plasmids** | *p*ET28a (+) | Expression vector (5369 bp); KmR, T7 promoter, His-Tag | Promega Co., USA |
| **Phages** | Staphylococcus phage vB_SauR_SW21 | Accession No.OR683639 | (22) |
| **Primers** | Forward primer: | ^5’^GCGCGCCCATGGGGAAATCACAACAACAAGCGA^3’^ | This study |
|  | Reverse primer: | ^5’^GCGCGCCTCGAGTGAGAACACCCCCCACGGAAT^3’^ | This study |
