# Supplementary material for: Antimicrobial and antibiofilm activity of a novel bacteriophage endolysin (LysSW21) against methicillin-resistant Staphylococcus aureus
Source: BMC Microbiol. 2026 Apr 16;26:507. doi: 10.1186/s12866-026-04916-w (PMC13214339; doi:10.1186/s12866-026-04916-w)
Supplement: Supplementary file 3 — Supplementary Material 3. [file 12866_2026_4916_MOESM3_ESM.docx]

**Table 2. Physicochemical features of LysSW21 protein.**

| **Physicochemical characteristics** | |
| --- | --- |
| **Formula** | C_1297_H_1879_N_333_O_380_S_8_ |
| **Number of amino acids** | 249 |
| **Total number of atoms** | 3897 |
| **Theoretical pI** | 5.81 (acidic) |
| **Molecular weight** | 28.47 kDa |
| **negatively charged residues (Asp + Glu)** | 24 |
| **positively charged residues (Arg + Lys)** | 21 |
| **Instability index** | 23.58 (Stable) |
| **Aliphatic index** | 60.64 |
| **Grand average of hydropathicity (GRAVY)** | -0.559 (hydrophilic) |
